# Cell Fate Decision Making through Oriented Cell Division

**DOI:** 10.3390/jdb3040129

**Published:** 2015-12-14

**Authors:** Evan B. Dewey, Danielle T. Taylor, Christopher A. Johnston

**Affiliations:** Department of Biology, University of New Mexico, Albuquerque, NM 87131, USA

**Keywords:** cell fate determinants, cortical polarity, spindle orientation

## Abstract

The ability to dictate cell fate decisions is critical during animal development. Moreover, faithful execution of this process ensures proper tissue homeostasis throughout adulthood, whereas defects in the molecular machinery involved may contribute to disease. Evolutionarily conserved protein complexes control cell fate decisions across diverse tissues. Maintaining proper daughter cell inheritance patterns of these determinants during mitosis is therefore a fundamental step of the cell fate decision-making process. In this review, we will discuss two key aspects of this fate determinant segregation activity, cortical cell polarity and mitotic spindle orientation, and how they operate together to produce oriented cell divisions that ultimately influence daughter cell fate. Our focus will be directed at the principal underlying molecular mechanisms and the specific cell fate decisions they have been shown to control.

## 1. Introduction

The ability of multicellular organisms to specify a vast diversity of cell fates from a single zygotic origin is a truly remarkable and fascinating biological feat. Identifying the mechanisms of cell fate specification is fundamental to understanding animal development, as is defining how wrong decisions are made that lead to disease. Across metazoan taxa, cells have acquired the ability to orient their divisions with respect to a defined polarity axis. In many cases, this highly coordinated event occurs through both intrinsic and extrinsic cues that direct mitotic orientations so as to bias the separation of cell fate determining factors into specific daughter cells. The relationship between cortical cell polarity and the orientation of the mitotic spindle instructs cell fate decisions that are critical for tissue development and homeostasis. Emerging evidence portends a link between defective oriented cell divisions and a range of human diseases, including cancer [1,2]. In this review, we will highlight key cell polarity and spindle orientation complexes and discuss how oriented cell division controls cell fate acquisition across diverse tissues.

## 2. Cell Polarity Complexes Controlling Cell Fate Decisions

### 2.1. The Par/aPKC Complex

The evolutionarily conserved Par complex is pivotal to the establishment of cortical cell polarity. This complex includes three key proteins: atypical protein kinase C (aPKC), partitioning defect 3 (Par-3, a.k.a. Bazooka in *Drosophila*), and partitioning defect 6 (Par-6). Together, these proteins work in a variety of different cellular contexts throughout development across many organisms to regulate cortical cell polarity.

**Figure 1:**
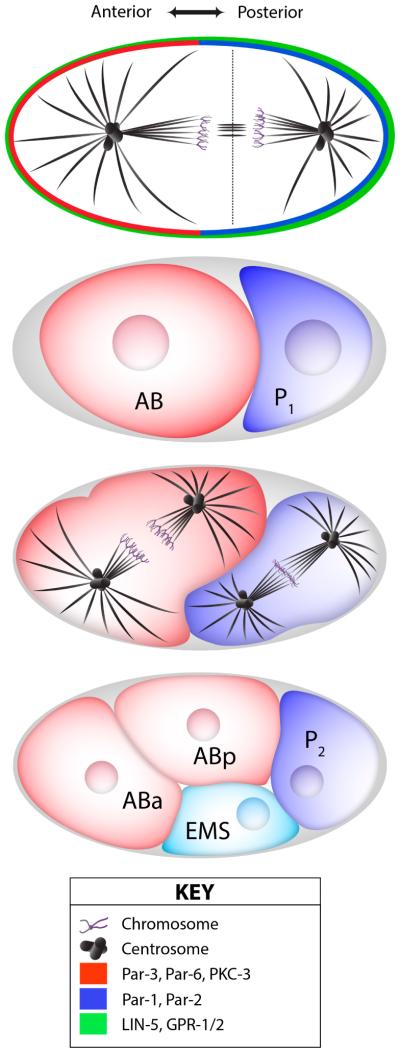
Oriented cell division in the *C. elegans* zygote. The first zygotic division in *C. elegans* proceeds asymmetrically to generate differential AB and P1 cells. Par proteins in the zygote are polarized along an anterior-posterior cortical axis: the anteriorally-localized Par-3/Par-6/aPKC (**red**) and posteriorally-localized Par-1/Par-2 (**blue**) complexes mutually repress cortical localization of one another. Spindle orientation along this polarity axis is regulated by the GPR-1/2 and LIN-5 complex (which is enriched at the posterior cortex), ensuring proper asymmetry in polarity protein distribution in daughter cells. This complex also induces a physical, posterior displacement of the spindle apparatus relative to the cell center, thereby generating a size asymmetry in offspring. Spindles in the resulting AB and P1 cells rotate relative to the original zygotic axis in subsequent divisions, yielding further diversification at the four-cell stage. These cells ultimately lead to the production of distinct cell lineages and their associated tissue structures in the developing animal [11].

Early studies in model organisms demonstrated that aPKC activity was critical for regulation of cortical polarity. In the *C. elegans* zygote, the Par complex was found to promote polarity along the anterior-posterior (A-P) axis in the zygote [3–5] (Figure 1). Upon fertilization, a breaking of symmetry initiates a “cortical flow” using contractile actomyosin forces to mediate movement of the anterior Par genes (aPKC/Par-3/Par-6) to the anterior side of the cell [4,5]. The posterior Par genes (Par-1/Par-2/Lgl [lethal giant larvae]) are initially present on the posterior side but expand along the posterior cortex with the help of Par-2 phospholipid binding activity, as well as positive feedback through membrane recruitment of cytoplasmic Par-2 by membrane bound Par-2 [6,7]. Once polarity has been established, phosphorylation by members of both the anterior and posterior Par genes function to maintain a mutually exclusive A-P boundary [6,8] (Figure 1). The serine/threonine kinase Par-1 functions to restrict the anterior members via phosphorylation of Par-3, while the kinase activity of aPKC functions to restrict anterior members via phosphorylation of Par-2 and Lgl [8–10]. Polarization of the embryo functions to produce distinct cell types by segregation of cell fate determinants upon oriented divisions [11] (Figure 1). Allotment of these determinants codifies the body structure of the mature animal, with many determinants functioning as cell cycle regulators, transcription factors, and components of cell trafficking complexes to achieve and maintain the final body pattern (for a more extended review of these functions see [12]). Without proper polarity, restriction of cell fate determinants and thus development of the animal are compromised. In embryos that are deficient of myosin, Par-6 distribution to the anterior cortex is compromised, indicating a requirement of cortical flow [5]. The Par genes themselves are also required for cortical flow, as embryos lacking Par-3 and Par-6 are deficient in this activity [3–5].

**Figure 2:**
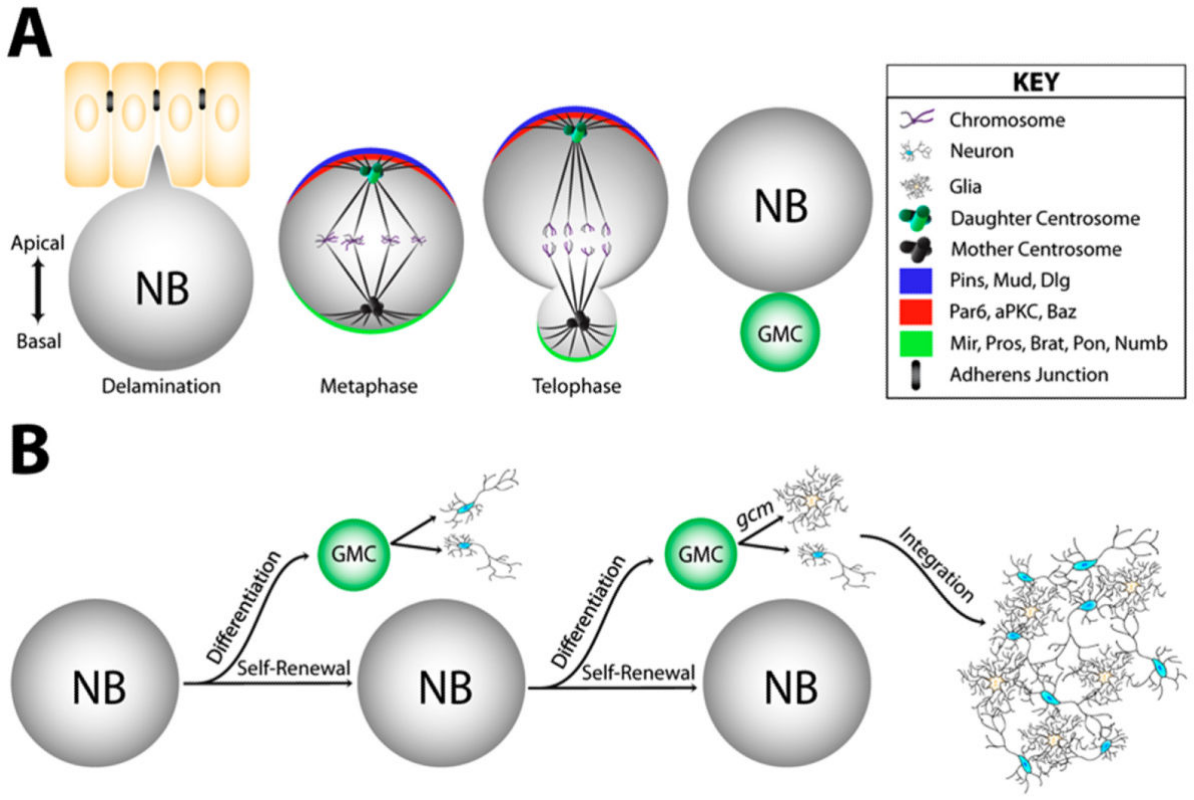

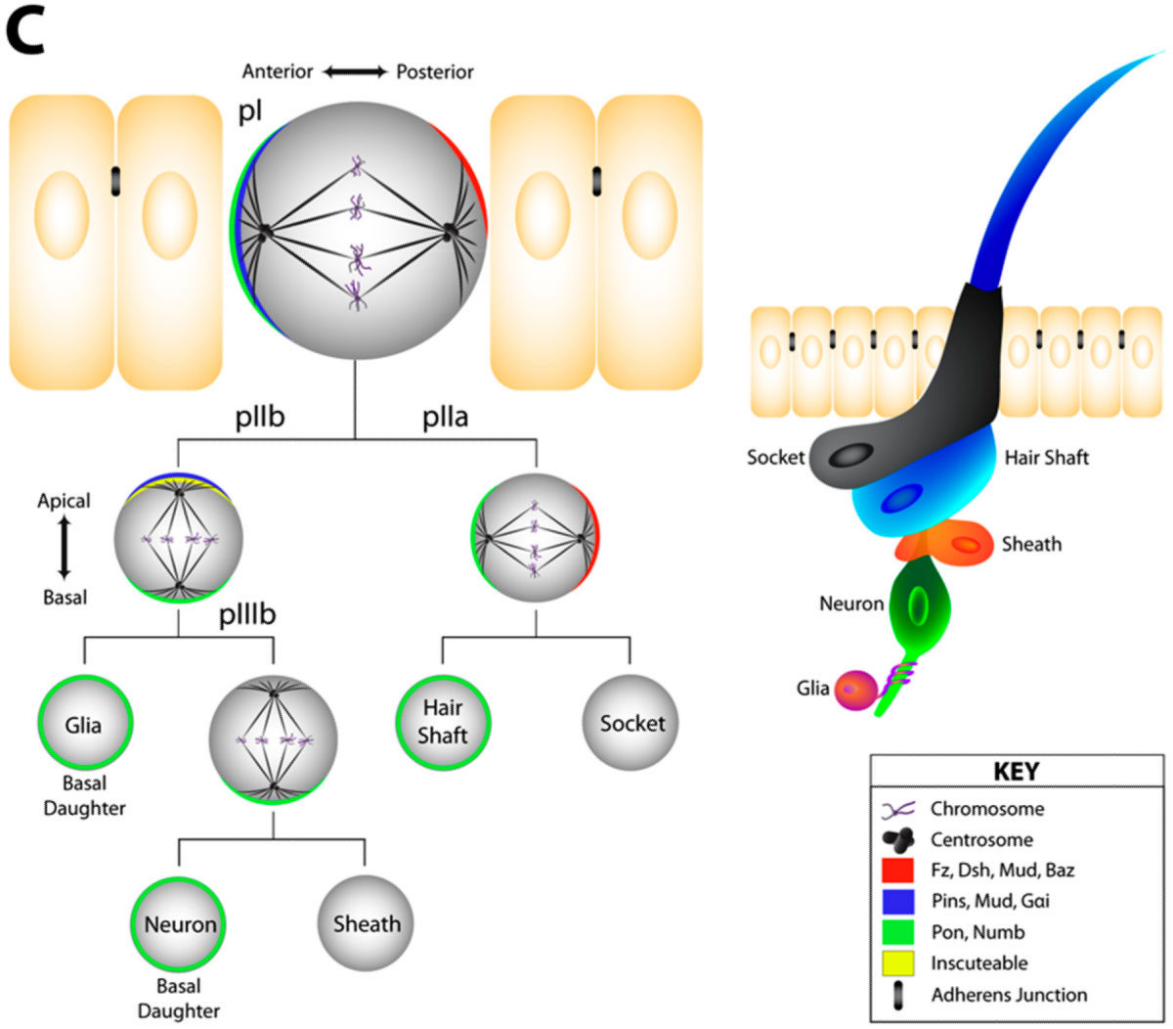
Oriented cell division in *Drosophila*. (**A**) Neuroblasts delaminate from the neuroepithelium and establish and apical-basal cortical polarity. The stemness-promoting aPKC enzyme (red) is apically localized and functions to restrict differentiation complexes (green) to the basal cortex. Spindle orientation along this polarity axis is achieved through the function of the apical Pins/Mud/Dlg complex (blue). In addition to asymmetric protein distribution, centrosomes are asymmetrically inherited in daughter cells with the daughter and mother centrosome preferentially segregated in the self-renewed neuroblast and differentiating ganglion mother cell (GMC), respectively; (**B**) Neuroblast divisions must properly balance self-renewal with differentiation: GMC daughters generate neurons and glial cells that integrate into the functioning central nervous system, whereas the neuroblast pool remains relatively constant throughout development. *gcm (*glial cells missing*)* is a gene that is known to regulate glial cell fate in Drosophila. If *gcm* is upregulated in a GMC daughter cell, it will differentiate into a glial cell [28,29]; (**C**) Sensory organ precursors (SOPs) produce mechanosensory organs within the developing wing. The initial pI cell polarizes along an anterior-posterior axis and uses two distinct spindle positioning pathways, anterior Pins/Mud and posterior Fz/Dsh, to asymmetrically distribute Numb to the anterior pIIb daughter. Further rounds of oriented divisions, including an Insc-mediated apical-basal division of the pIIb, results in the mature mechanosensory organ structure consisting of five distinct cell fates that function in concert within the adult wing tissue.

In *Drosophila* neuroblasts, the Par complex functions to polarize the cell along an apical-basal (A-B) axis, wherein neuronal differentiation factors are restricted opposite Par proteins to the basal cortex [13–15] (Figure 2A). Establishment of apical polarity is mediated by the interaction of Par-6 with the membrane anchored GTPase Cdc42 and may also be mediated by Par-3 interaction with phopshoinositides [16–18]. The neuronal fate proteins Numb and Miranda (Mir) both define basal polarity. Mir additionally functions to localize the cell fate determinants Prospero (Pros) and Brain tumor (Brat) to the basal membrane [19,20]. Basal Numb targeting involves its interaction with the transmembrane domain protein Moladietz (Mol, a.k.a. Numb interacting protein) as well as Partner of Numb (Pon), while Mir localization appears to depend on interaction with Myosin VI [21–23]. Similar to function in *C. elegans*, the Par complex maintains polarization through aPKC phosphorylation, restricting basal proteins Numb and Mir from crossing onto the apical membrane [24,25]. To prevent aPKC activity (and resulting loss of Mir) on the basal cortex, the protein Lethal giant larvae (Lgl) functions to inhibit aPKC basally [14,26]. Par-3 appears to be the most upstream component for apical polarity, as loss of Par-3 results in loss of all three Par complex components from the membrane, while Par-3 remains apically polarized with the loss of either aPKC or Par-6 [15]. Loss of Mir leads to loss of both Pros and Brat from the basal cortex, as well as and over proliferation of neuroblasts due to lack of fate determinant segregation, a phenotype also observed with loss of Numb [19,20,27].

In the mammalian context, several important aspects of Par complex function have been uncovered using an elegant three-dimensional tissue culture system with MDCK luminal cysts. In these cells, the Par complex is required for generation of the A-B axis and efficient lumen formation [30–34]. Initiation of lumen formation is dependent on three interdependent events: Par-3 and aPKC localization to the apical membrane initiation site (or AMIS), efficient delivery of Rab8/11-positive apical vesicles to this site, and initiation of a GTPase cascade by apical vesicular protein Rab11a to drive both vesicular delivery and localization of the Par complex [30]. These apical vesicles deliver the GTPase Cdc42, which (in conjunction with the Par complex) is crucial to initial expansion of the AMIS to the pre-apical patch (PAP) and for maintenance of the eventual apical membrane [30,35]. Further, it has been found that phospholipids become polarized via the Par complex in this process, with phosphotidyinositol-(4,5)-bisphosphate becoming apically localized and phosphotidyinositol-(3,4,5)-trisphosphate becoming basolaterally localized [36–39]. This is mediated by apical localization the lipid phosphatase PTEN, with binding to Par-3 contributing to this activity. In line with this activity for the Par complex, aPKC has been shown to mediate (PI(4,5)P2) asymmetric localization in MDCK monolayer development [40]. After polarity establishment, aPKC phosphorylation of Par-3 localizes it to tight junctions, providing a physical barrier between the apical and basolateral membranes to restrict protein localization [31,32,34]. In addition to this activity, aPKC also phosphorylates key spindle orientation components (see below) to restrict them basolaterally and maintain lumen integrity [41,42]. Cysts with loss of aPKC, Cdc42, or Par-3 all result in formation of multiple lumens, indicating the necessity of Par complex members in regulation of this process [30,32].

### 2.2. The Notch/Numb Pathway

Notch signaling is an essential developmental signaling cascade in multicellular animals, participating in a multitude of cellular processes. Notch is a single transmembrane receptor that is activated by Delta, another transmembrane protein, through direct cell-cell interaction. Delta-Notch interaction promotes proteolytic cleavage of the intracellular tail of Notch (Notch^INTRA^) by the γ–secretase complex, ultimately leading to the regulation of gene transcription. Notch activity can be inhibited by Numb, an intracellular protein, and differential Numb expression can produce disparate levels of Notch signaling [43]. Although Notch signaling participates in a host of cellular activities, a notable function is in the determination of cell fates.

In *Drosophila* neuroblasts, Numb is restricted to the basal cell cortex by suite of apical polarity proteins, including both the Par/aPKC and Lgl/Discs large (Dlg) complexes [14,44] (Figure 2A). This polarized localization allows for selective Numb segregation into the ganglion mother cell (GMC) daughter where it promotes differentiation through inhibition of Notch. Asymmetric Numb localization is dependent on the apical polarity complex, including the activity of Dlg and Lethal giant larvae (Lgl) [45,46]. Loss of Lgl or Dlg causes Numb mislocalization, resulting in an expansion in the number of neuroblasts populating the larval brain [14]. Asymmetric Numb segregation is also critical for proper cell fate specification during the development of mechanosensory bristle organs in the fly wing [47] (Figure 2C), wherein Numb inhibition of Notch signaling is essential for proper cell fate specification during mechanosensory organ maturation [48]. Specifically, individual pI sensory organ precursor (SOP) cells are multipotent cells that give rise to five distinct cell fates that collectively function as a single bristle structure capable of sensory perception (Figure 2C). Numb is localized at the anterior cortex of the pI cell and subsequently asymmetrically segregated into the pIIb daughter. Here, Numb promotes endocytosis of Notch in order to down-regulate signaling specifically in the pIIb daughter of the pI division [49]. Mitotic pIIb cells localize Numb at the basal cortex and segregate it specifically into what will differentiate into the glial cell of the organ [47]. Thus, distinct Numb asymmetries can be achieved across multiple cell divisions to regulate cell fate specification.

Cortical polarity is a fundamental aspect of epithelial cell structure and function. Within their respective tissue, epithelial cells are tightly adhered together via cell-cell junctions that demark a cortical polarization defined by apical and basolateral domains. Concentrated aPKC localization at cell-cell junctions inhibits apical localization of Numb, thus sequestering it to the basolateral domain. This mutually exclusive localization is achieved by direct, aPKC-mediated Numb phosphorylation, which is thought to electrostatically repel Numb interaction with membrane phospholipids and is likely an evolutionarily-conserved mechanism of polarity establishment [25]. As Numb is implicated in the recycling of membrane proteins [50,51], polarized Numb localization could provide a means for spatially restricted endocytosis at the basolateral surface.

Development of the neural tube in vertebrates, a process termed neurulation, involves folding of the neural plate followed by patterned cell fate acquisition. In neuroepithelial cells, members of the prototypical Par/aPKC complex become apically enriched prior to the onset of neurulation [52]. During subsequent events in the neurulation process, Numb localizes asymmetrically to the basolateral domain, consistent with a model of aPKC-dependent Numb polarization [53]. Numb localization depends on its N-terminal domain, and mutations that diminish the protein interaction capacity of this region result in defective convergence and extension morphogenic events as well as neural tube development [54]. Mammalian neurogenesis also requires asymmetric Numb signaling regulated through Par polarity mechanisms, ultimately regulating the balance in daughter cell fates and proper cortical development [55–57].

## 3. Spindle Orientation Complexes Controlling Cell Fate Decisions

**Figure 3:**
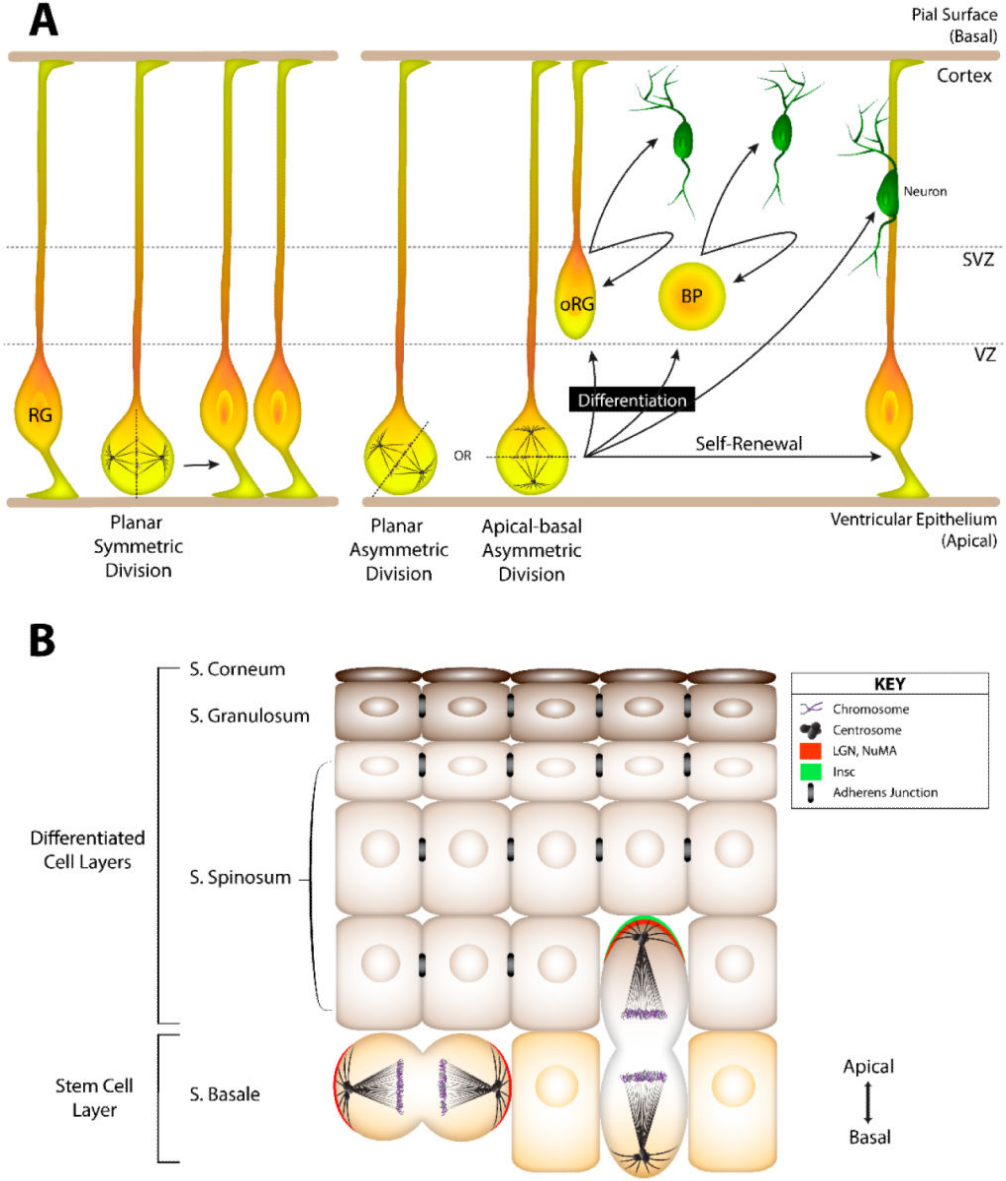
Oriented cell division in mammals. (**A**) Neurogenesis depends on properly balanced modes of division within radial glial progenitor cells (RG) within the ventricular zone (VZ). Planar symmetric divisions yield two RG cells, whereas asymmetric divisions drive differentiation within the subventricular zone (SVZ) and cortex. These asymmetric divisions are associated with altered spindle orientation relative to the overlying epithelium and produce outer RG (oRG), basal progenitors (BP), or neuron cells; (**B**) The mouse epidermis also relies on balanced output in mitotic symmetry for development of several differentiated layers. Keratinocyte stem cells in the basal layer undergo symmetric divisions in order to promote tissue growth and expansion. Insc expression induces an apical-basal orientation of cell division that allows for differentiation necessary for tissue stratification. The LGN/NuMA complex is critical for maintaining proper spindle orientation during this process.

Once cortical polarity has been established, the ability to reliably segregate polarized cell fate determinants differentially into respective daughter cells is mandated if asymmetric fate specification is to be achieved. Asymmetric fate inheritance occurs pursuant to a cleavage furrow ingression site that results in cytokinesis perpendicular to the polarity axis (Figures 1–3). Because the mitotic spindle equator marks the site of contractile ring formation [58], proper spindle alignment along the polarity axis plays an important role in cell fate specification. Several recent reviews have thoroughly detailed an impressively diverse set of spindle positioning pathways and the pathways through which they communicate with the spindle apparatus [59–63]. For brevity, we will restrict our discussion to two well-defined spindle orientation complexes, both of which have intricate links to cortical polarity systems that control cell fate specification.

### 3.1. The Pins/Mud/Dlg Complex

**Figure 4:**
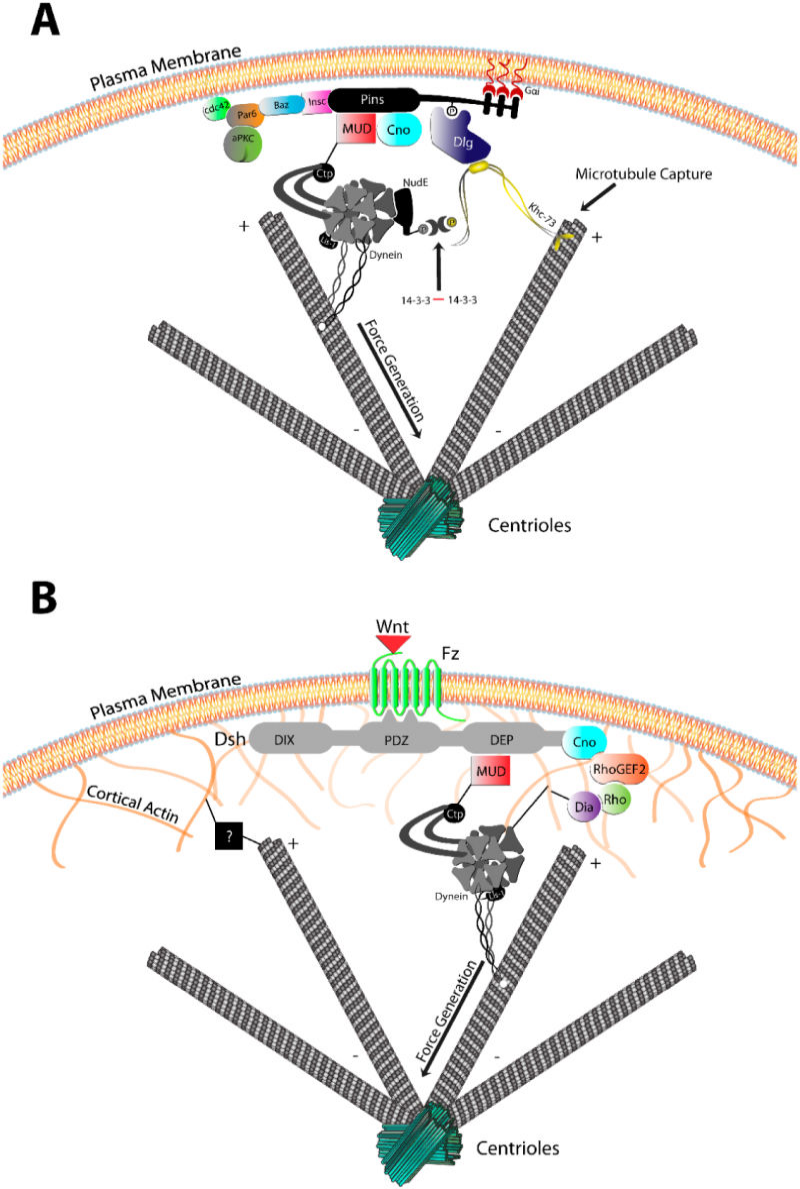
Molecular models of spindle orientation complexes. (**A**) Pins mediates spindle orientation through two synergistic pathways: a microtubule-capturing complex consisting of Dlg/Khc-73 and a force-generating complex consisting of Mud/Dynein. These two pathways are linked through a 14-3-3 dimeric bridge; (**B**) Fz/Dsh also utilizes two pathways to position the mitotic spindle: the Mud/Dynein complex likely provides an analogous force-generating function, whereas the Dsh C-terminus initiates cortical F-action polymerization through Rho/Diaphanous (Dia) that might serve a spindle capturing role through an unknown mechanism.

Perhaps the most well-characterized spindle orientation complex is that assembled through the cortically-localized scaffold protein Partner of Inscuteable (Pins) (Figure 4A). *Drosophila* Pins has evolutionarily conserved orthologs in worms (GPR1/2) and mammals (LGN) that, moreover, serve orthologous functions as spindle orientation regulators [64]. Cortical localization of Pins is dependent upon Inscuteable (Insc), a protein that also associates with the Par polarity complex [65,66]. Interestingly, Insc-mediated localization of Pins can be induced at specific developmental time points in order to signal a shift to asymmetric cell divisions. For example, in the neuroepithelium of the *Drosophila* optic lobe, expression of Insc induces apical Pins polarity and is associated with a switch to asymmetric divisions that yield a delaminated daughter cell that adopts a neuroblast fate [67]. A similar scenario occurs in the mouse epidermis in which Insc-mediated Pins polarization induces a switch from symmetrically dividing keratinocytes to asymmetric divisions critical for tissue stratification (Figure 3B). Loss of Pins in these cells prevents fate transition, leading to underdeveloped skin tissue defective in proper fluid and electrolyte maintenance [68]. Pins-mediated spindle orientation is also influential in asymmetric division of *Drosophila* neural stem cells and mechanosensory hair cells [65,69,70] (Figure 2), the first zygotic division of developing *C. elegans* [71–73] (Figure 1), and mammalian cerebral neurogenesis controlled by oriented division of progenitor cells [74–76] (Figure 3A). Thus, Pins regulates spindle positioning within diverse cells and across evolutionary time.

The molecular machinery through which Pins directs spindle positioning has been elegantly illuminated over the past decade. Pins activity relies on its ability to organize microtubule-associated motor proteins that influence the dynamics of the mitotic spindle [60]. Initial studies demonstrated a role for the minus-end directed motor cytoplasmic dynein downstream of Pins. Together with the Dynactin complex [77,78], Dynein exerts cortical pulling forces on spindle microtubules that are critical not only for precise alignment with Pins [79–81], but in systems such as the *C. elegans* zygote this unequal cortical force also physically displaces the spindle along the polarity axis to induce daughter cell size asymmetry [71,72] (Figure 1). Studies in cell culture have nicely demonstrated that cortical Dynein is likely sufficient for the force generation aspect of Pins function [82]. Pins association with the dynactin/dynein complex is indirect, relying on a key adaptor protein called Mushroom body defect (Mud), as well as possible other unknown components. Pins and Mud directly interact, and Pins is required for cortical Mud localization, which, in turn, is necessary for subsequent dynein activation [79,81]. Loss of Pins, Mud, or dynactin/dynein all perturb proper spindle orientation. Elegant fate tracking experiments in *Drosophila* neuroblasts have demonstrated that loss of spindle orientation alone (through loss of Mud expression) can result in improper cell fate specification, specifically by expanding the stem cell pool [83]. Furthermore, loss of Pins is synthetic with loss of the polarity gene Lethal(2) giant larvae (Lgl) in *Drosophila* neuroblasts leading to massive stem cell overgrowth and brain tumors with invasive capabilities upon implantation in wild-type host flies [14]. These studies illustrate the importance of Pins/Mud-mediated spindle orientation in cell fate acquisition and may suggest a tumor suppressor activity in stem cells [84].

Subsequent studies identified a role for a second pathway downstream of Pins during spindle positioning. Again using *Drosophila* neuroblasts as a model, Siegrist and Doe identified a role for the tumor suppressor protein Discs large (Dlg) [85] (Figure 4A). Dlg directly binds Pins but only after Pins has been phosphorylated by the mitotic kinase Aurora-A, highlighting a temporal link with cell cycle progression [80,86]. Pins/Dlg association has subsequently been shown to be important for spindle positioning in *Drosophila* epithelia and chick neuroepithelium [87,88]. Association with Dlg is necessary for subsequent binding and activation of a second microtubule motor, the plus-end directed kinesin Khc73 [85,89]. The function of this Dlg/Khc73 complex is two-fold. First, plus-end trafficking serves as a mode of microtubule-induced Pins polarity establishment [85]. Secondly, microtubule association of polarized Pins/Dlg/Khc73 serves as a capture site for dynamic astral microtubules that appears to initiate the spindle orientation process [80]. Subsequent Pins/Mud/Dynein-mediated forces complete the alignment process, resulting in synergistic function of the two motor-based pathways. Interestingly, a recent study demonstrated further collaboration between these Pins pathways in which Dynein and Khc73 are physically linked by a NudE/14-3-3 complex [90]. Thus, Pins permits accurate spindle orientation through a complex assembly of dual acting microtubule motors that cooperate to achieve maximum fidelity.

The molecular mechanism of Pins-mediated spindle orientation remains a vigorously-investigated topic, with several important findings having emerged even within the last year. Two groups independently discovered a novel intersection between Aurora-A and Lgl [91,92]. In epithelial cells, phosphorylation of Lgl by Aurora-A results in cortical release into the cytoplasm. Preventing cortical release of Lgl during mitosis disturbs spindle orientation within the tissue plane, possibly through Lgl/Dlg binding that competes with Pins/Dlg complex formation. Interestingly, whereas PKC phosphorylation of Lgl is necessary only for cortical polarity, Aurora-A activity specifically controls spindle orientation. These studies also revealed that Lgl not only regulates asymmetric cell divisions but also symmetric mitoses of epithelia. Several recent studies have also illuminated a link between the Pins/Mud complex and Sterile20-like kinases (Ste20). Ste20 kinases are evolutionarily conserved serine/threonine kinases that regulate cell polarity and proliferation [93]. Using cultured mammalian cells, Machicoane *et al.* recently demonstrated that Ste20-like kinase (SLK) phosphorylates ezrin/radixin/moesin (ERM) proteins, which participate in linking cell membranes to the underlying actin cytoskeleton, to ensure proper spindle orientation. This effect was attributed to promoting and sustaining the cortical association between LGN and NuMA [94]. Even more recently, two independent studies identified a role for Hippo and Warts kinases, two additional members of the Ste20-like family, in spindle orientation. In *Drosophila* neuroblasts, Warts associates with the Par polarity complex and phosphorylates Canoe, a Mud-interacting protein, to promote Pins-dependent spindle orientation and asymmetric stem cell divisions [95]. In *Drosophila* wing disc epithelial cells, Warts phosphorylation of Mud is essential for cortical association with Pins to ensure planar spindle orientation [96].

### 3.2. The Frizzled/Dsh Complex

Wingless/Wnt signaling represents one of the most extensively studied developmental signaling pathways. Graded distributions of Wnt secretion provide a non-autonomous signal that establishes planar cell polarity underlying the orientation of tissue axis [97]. Wnt also provides an important directional cue for morphogenic events such as gastrulation and convergent extension during development [98]. Finally, Wnt-dependent transcriptional regulation determines cell fate by promoting stemness in several tissues, including intestinal crypts [99].

The ability of Wnt to orchestrate tissue-wide planar cell polarity relies on the polarization of its receptor Frizzled (Fz) and proximal signaling effector Dishevelled (Dsh). In addition to its effects on cell polarity, the Fz/Dsh complex also acts as a vital spindle orientation complex across diverse tissues and species (Figure 4B). Initial discoveries of Fz/Dsh as a spindle orientation cue involved pI SOP cells. Here, Fz/Dsh serves as an important anterior-posterior spindle positioning cue to ultimately separate Numb asymmetrically into the pIIb daughter. Fz/Dsh mutants have defective mechanosensory organ development and abnormal adult hairs [47,100,101]. Similar phenotypes occur in mammalian systems, demonstrating the conserved nature of this Fz/Dsh function [97]. Fz/Dsh also participates in oriented divisions of *C. elegans* neuroblasts [102] and zebrafish dorsal epiblasts [103].

The molecular pathways linking Fz/Dsh to spindle orientation have recently been investigated (Figure 4B). Interestingly, despite a lack of predicted sequence and structural homology to Pins, Dsh also associates with the Mud/Dynein complex as one necessary link to the mitotic spindle. Loss of Mud prevents Fz/Dsh-mediated spindle orientation in *Drosophia* SOPs as well as during zebrafish gastrulation [104]. The precise atomic details of the Dsh/Mud interaction remain to be elucidated, but appears to involve the central DEP domain in Dsh. Although less clear than studies involving Pins, engagement of Dynein activity through Mud association is likely to afford a similar force generating mechanism during spindle positioning. Unlike Pins, however, Dsh does not require Dlg/Khc73 activity. Instead, non-canonical Fz/Dsh signaling induces an asymmetric cortical actin “cap” through activation of the formin F-actin nucleating protein Diaphanous (Dia) [103,105]. How this Dsh-induced cortical actin couples to spindle microtubules has not been resolved, although actin has long been implicated in spindle orientation in numerous systems [106]. One possibility would be that microtubule-binding myosin motors (those containing MyTH4 domains) exert spindle oriented forces along the Dia-induced linear actin cables at the site of Fz/Dsh polarity. Such myosin proteins are involved in centrosome migration and orientation [107,108]. Cytoskeletal crosslinking proteins are another attractive candidate that could provide a microtubule capturing mechanism at the actin cap akin to the Dlg/Khc73, possibly by reducing the dynamic instability of astral microtubules upon capture at the actin cortex [109,110]. Interestingly, chimeras combining Pins/Mud and Dsh/Dia elements or, vice versa, the Dsh/Mud and Pins/Dlg elements retain full function, demonstrating remarkable modularity in spindle orientation pathways [105]. Furthermore, the Pins and Dsh pathways are known to coordinate activities in certain cases [104], and physical interactions between components provide the possibility of direct pathway crosstalk [111].

Although not as extensively studied as the Pins complex, details of the molecular basis for Dsh-mediated spindle orientation have continued to emerge. An intriguing recent study found that deubiquitination of cortical Dsh by cylindromatosis (CYLD) contributed to its association with NuMA and the dynein/dynactin complex [112]. CYLD also stabilizes astral microtubules of the mitotic spindle, which further promotes activity with the cortical Dsh/NuMA during spindle positioning. These findings highlight a role for an additional post-translational modification in spindle positioning, together with the more appreciated role of phosphorylation discussed previously. Another recent study in *C. elegans* investigated the role of cell contacts in Dsh function. In the ABar and EMS cell divisions (see Figure 1), mitotic spindles reorient relative to the zygotic division in response to Wnt signaling through cortically enriched Dsh. Dejima *et al.* found that syndecan (SDN-1), a member of the heparin sulfate proteoglycan family, was responsible for the asymmetric localization of Dsh at the interface between these cells. SDN-1 was specifically required for spindle orientation in the ABar cell [113]. These recent findings collectively extend our understanding of how polarized Wnt signaling can communicate with spindle microtubules during oriented cell divisions.

## 4. Cell Fate Decisions Made Through Oriented Cell Divisions

Establishment of cortical polarity and orientation of the mitotic spindle are two fundamental components of daughter cell fate decisions. Rather than operating as independent steps in this process, these two operations are coordinated to ensure proper fate specification. Below we discuss several specific examples of how oriented cell division, through the linking of cell polarity and spindle positioning, dictates daughter cell fates and the possible consequences of this process gone wrong.

### 4.1. Cell Fate Decisions in the Developing Brain

*Drosophila* neural stem cells (neuroblasts) are an excellent model system for asymmetric cell division—neuroblast divisions are asymmetric by both size and molecular identity, yielding a larger self-renewed neuroblast and smaller ganglionic mother cell (GMC) specified for neuronal differentiation (determined by asymmetric inheritance of Numb and other cell fate determinants) (Figure 2B). In this paradigm, a relatively small number of neuroblasts can supply the vast number of differentiated progeny that populate the central nervous system. Neuroblasts utilize the Par/aPKC complex to establish an apical-basal polarity axis; co-localization of the Pins/Mud/Dlg complex at the apical cortex serves as the prominent spindle positioning cue [114]. While the size asymmetry likely involves a complex combination of poorly understood processes [115], the molecular asymmetry is well understood to rely on unequal segregation of stemness promoting factors (e.g., aPKC) to the apical neuroblast and differentiation specifying factors (e.g., Numb, Pros, Brat) to the basal GMC. Loss of polarity genes such as aPKC and Lgl have been shown to induce a modest increase in stem cell numbers, whereas combined loss of polarity and spindle orientation components (e.g., Lgl and Pins double mutants) shows a remarkably synthetic phenotype characterized by severely overgrown brains and dramatic neuroblast expansion [14]. Moreover, these neuroblast-rich tissues display invasive growth and features of metastasis when explanted in wild-type hosts, demonstrating that proper execution of oriented cell division ensures proper maintenance of stem cell proliferation [84]. Conversely, loss of the Lis-1 gene, an activator of dynein motor activity and component of the spindle orientation machinery [77,80], causes loss of neural stem cell proliferation and has been linked with the neurodevelopmental disorder, Primary Lissencephaly [116].

Because the polarity and spindle orientation complexes are intimately connected, discerning the exact role of spindle orientation itself on cell fate determination has been problematic. One study took advantage of Mud mutant neuroblasts that retain all known polarity markers intact while displaying marked defects in spindle orientation. By tracking multiple rounds of cell division, it was discovered that equal segregation of apical polarity complexes consistently resulted in two daughter cells with neuroblast fate; only forced overexpression of the basal determinant Pros could override neuroblast identify in favor of differentiation [83]. Interestingly, however, Mud mutant brains show only a modest increase in total neuroblast numbers and do not severely overgrow, suggesting that spindle orientation is required for balancing stem cell fate specification but not necessarily sufficient for inducing tumorigenic phenotypes. This fits with other prevailing models suggesting spindle orientation provides a tumor suppressor mechanism but itself is not a sufficient route to tumor development [84].

Neurogenesis in the mammalian cerebral cortex also relies on properly oriented cell divisions, wherein neural stem cells must balance proliferative symmetric divisions, which occur predominantly during early developmental phases, against diversity yielding asymmetric divisions that ensue later in neurogenesis [75] (Figure 3A). The transition to asymmetric divisions is accompanied by a shift in spindle orientation from planar to oblique/vertical relative to the basal domain, ultimately affecting inheritance patterns Notch signaling regulators [117]. Although additional complexities such as multiple progenitor cell types can confound assumptions of direct causal links between spindle orientation and cell fate in this system, defects in a number of known spindle orientation regulators are correlated with altered cell fate acquisitions [118–120]. However, cell fate switching can occur in the absence of oblique spindle reorientation as well [76]. Several more recent studies demonstrated the existence of an intermediate progenitor fate (outer radial glia cells) that is acquired through the actions of the Insc/Pins spindle orientation complex [74,121,122]. These studies more firmly support a causality between spindle orientation and cell fate outcomes; disruptions of early planar orientations deplete the progenitor cell pool [123], whereas defects in later oblique divisions diminish neuron production [122]. Spindle orientation alone may not suffice for cell fate determination in this system, as asymmetric inheritance of Par-3 and Notch, which can occur independent of spindle orientation changes, plays an essential role [55,124]. Despite these idiosyncrasies relative to the *Drosophila* model, spindle orientation clearly contributes an important role to cell fate determination during mammalian neurogenesis.

### 4.2. Cell Fate Decisions in Epithelial Tissue

Epithelial tissue has proven another extremely useful model for investigating oriented cell division, and defects in this process may contribute to disruption of epithelial organization and promote tumorigenesis [125]. Cultured cells have been instrumental in defining models implicating the actin cytoskeleton and extracellular matrix in regulating spindle positioning through changes in cell shape and cortical rigidity [106,126–128]. Studies using three-dimensional cysts formed from individual epithelial cells have identified roles for the canonical Par polarity and Pins spindle orientation complexes in maintaining cell arrangements within defined structures [129]. In this system, Pins is localized along the lateral cell surface to ensure spindle orientation occurs parallel to the cyst lumen, preventing inappropriate luminal positioning of daughter cells that can induce disruption of the cyst geometry and eventual multi-lumen phenotypes [42]. This system is a promising *in vitro* proxy for events that may contribute to similar tissue architecture disruptions in polycystic kidney disease, a disease in which spindle misorientation has been implicated [130].

Several prominent *in vivo* models of oriented epithelial cell divisions have also been identified. The mouse epidermis is a stratified tissue containing multiple layers of differentiated cells providing specific functions. Keratinocyte stem cells reside in the basal layer and undergo two distinct modes of oriented division: spindle orientation parallel to the substratum results in a symmetric division, whereas orthogonal orientation provides a means of asymmetric cell division [68] (Figure 3B). Symmetric divisions provide a route for tissue growth and expansion, while asymmetric divisions yield differentiated progeny required for tissue stratification. As in the developing brain, this balance too is developmentally regulated on a temporal scale. At E17.5, cell divisions become primarily asymmetric in nature, a switch that is controlled by the expression of Insc and relocalization of LGN to the apical cortex to provide a cue for orthogonal spindle rotation [68,131]. Disrupting this homeostasis results in improper epidermal development and offspring incapable of proper solute and water transport in the skin [131].

The imaginal wing disc in *Drosophila* has been a workhorse genetic model for epithelial cell biology [132]. During epithelial mitosis, planar spindle orientation ensures that daughter cells are maintained within the tissue plane. Disrupting planar orientation is associated with epithelial-mesenchymal transition (EMT) not only during normal development [133], but may also contribute to diseases linked to inappropriate EMT such as cancer. Several recent studies have identified an essential link between spindle orientation and epithelial homeostasis using the wing disc model. Nakajima *et al.* found that planar spindle orientation is controlled by the Pins/Mud/Dlg complex and that disruptions in these genes (as well as others involved in cortical actin dynamics) upregulated JNK-dependent apoptosis. Simultaneous loss of spindle orientation and apoptosis induced tumor-like masses following EMT processes [134]. Poulton *et al.* reported similar findings, but also found that genes regulating centrosome integrity could upregulate apoptosis, leading to tissue underdevelopment [135]. Centrosome loss was associated with chromosome missegregation, spindle misorientation, and eventual cell death. Interestingly, a synthetic interaction was seen following loss of Sas-4 (centrosome loss) and either Pins or Mud (spindle misorientation), suggesting distinct roles for each of these events. The precise mechanism coupling spindle orientation and apoptosis pathways remains unknown, but these studies demonstrate an intriguing link that is consistent with a possible tumor suppressor role for spindle positioning [84].

The *Drosophila* follicular epithelium represents another established model for epithelial biology, in particular the study of cell polarity [136]. Early studies suggested that integrin signaling restricted spindle positioning within the tissue plane to prevent untoward stratification, a process that occurred independent of cell adhesion [137]. Rather, planar spindle positioning was found to occur through Pins/Mud- and Dlg-mediated pathways assembled along the lateral cell cortex [87]. By regulating both cell polarity and spindle orientation, Dlg appears to play a central role in oriented epithelial cell divisions. These results further demonstrate the remarkable diversity of the Pins spindle orientation complex.

### 4.3. Cell Fate Decisions in the Germ Line

**Figure 5:**
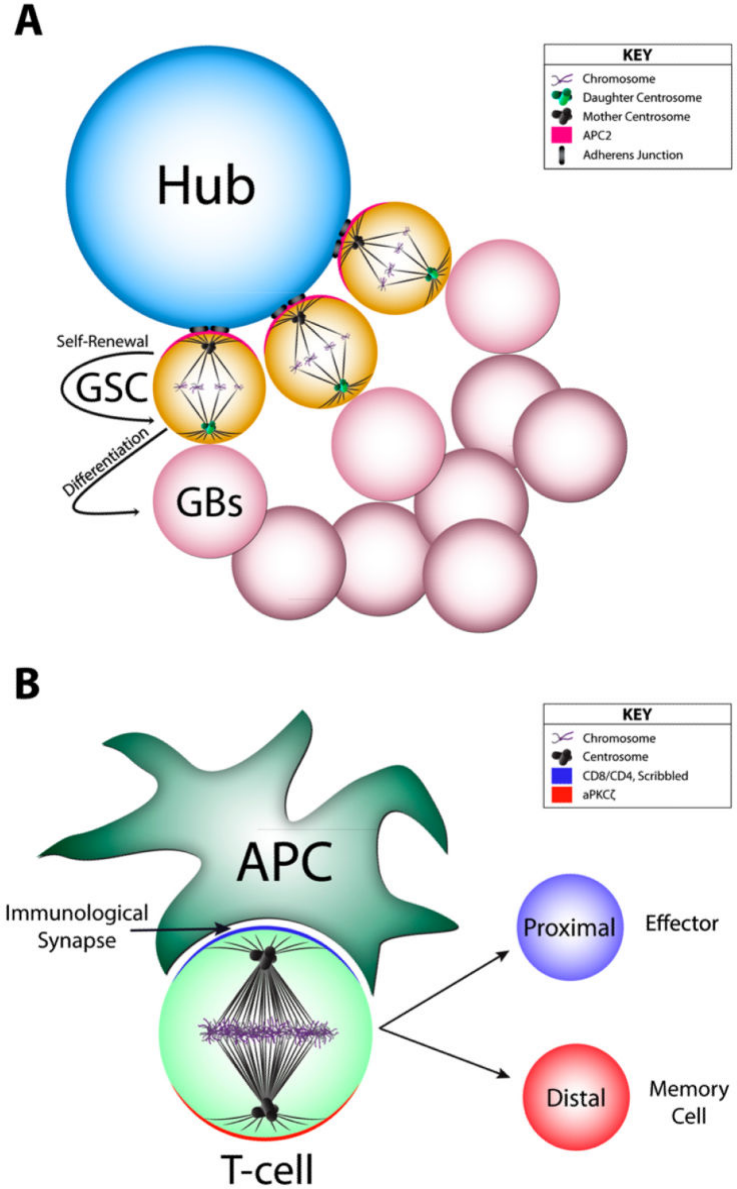
Spindle orientation to cell-adhesion cues. (**A**) *Drosophila* male germ line stem cells (mGSCs) form adherens junctions with a central hub cell and orient their mitotic spindles relative to this junction through the activity of the APC2 protein. Following division, the hub-proximal cell retains a stem cell fate, whereas the hub-distal cell becomes a gonial blast (GB) that differentiates further during spermatogenesis. Additionally, the GSC inherits the mother centrosome, while the daughter centrosome is segregated into the GB; (**B**) T-cell maturation initiates upon interaction with an APC at a specialized cell junction called the immunological synapse, mediated though interactions with CD8/CD4 receptors on the T-cell surface. APC interaction induces cell polarity in the T-cell, with Scribble (Scrib) localizing along the synapse and aPKC along the opposite cortical surface. Spindle orientation relative to the synapse generates a proximal and distal cell that differentiate into the Effector and Memory cell, respectively.

Spermatogenesis in *Drosophila* occurs through asymmetric division of male germ line stem cells (mGSCs). mGSCs decorate a central hub cell, an environmental niche, using cell-cell junctions and orient their mitotic spindle perpendicular to the hub, thereby generating a proximal self-renewed stem cell and a distal cell that undergoes differentiation [138] (Figure 5A). Stemness is promoted in the hub-proximal cell through activation of the JAK-STAT pathway by secreted Unpaired ligand from the hub [139,140]. Molecularly, spindle orientation is mediated through communication between an E-cadherin/Aramdillo complex (polarized at the hub-facing cortex) and the antigen-presenting cell (APC) tumor suppressor protein (localized at astral microtubule plus ends) [138]. More recently, it was discovered that Baz, a component of the Par complex, serves as a cue for centrosome position (cortical docking) prior to mitosis entry, which ultimately determines spindle orientation [141]. Although cadherin-mediated junctions have been implicated in other modes of spindle orientation [142], this pathway has been best defined in mGSCs and represents a rather unique and perhaps highly specialized mechanism relative to the Pins/Mud/Dlg complex.

### 4.4. Cell Fate Decisions in T-cell Selection

Clonal selection of T-cells produces both effector and memory cells necessary for the adaptive immune response to foreign antigens. T-cells can form a long-lived physical interaction with antigen-presenting cells (termed the “immunological synapse”), which induces polarity of signaling pathways through asymmetric actin polymerization [143] (Figure 5B). Upon antigen presentation, the lone pre-mitotic centrosome of naïve T-cells localizes in close proximity to components of the immunological synapse. Upon mitotic entry, this centrosome remains associated with the synapse, generating a bipolar spindle that aligns with respect to the T-cell polarity axis. The well-described polarity proteins Scribble and aPKCζ localize within and directly opposite the synapse, respectively [144,145]. Loss of aPKC in CD8^+^ T lymphocytes impairs fate specification and maturation, likely due to improper asymmetric cell division upon antigen presentation [144]. Ultimately, T-cell divisions proceed so as to asymmetrically segregate critical fate determinants in daughter cells, with CD8 and aPKCζ inherited by the synapse proximal and distal daughter, respectively. Expression marker profile of the CD8^+^ daughter suggests it assumes an effector cell fate, whereas the aPKC^+^ daughter expresses memory cell-specific markers [146]. Furthermore, loss of aPKC results in an increased percentage of symmetric divisions based on inheritance patterns of fate determinants such as IL-2Rα, IFNγR, and T-bet, ultimately leading to an imbalance in cell fate favoring overproduction of effector cells at the expense of memory cells [144]. Asymmetric divisions of memory CD8^+^ T-cells has also been suggested to play a role during rechallenge, although the molecular mechanisms are likely to differ from those described during initial infection [144,147,148]. Interestingly, the affinity of the T-cell receptor for the dendritic cell-presented antigen, along with the contact time between cells, plays an important regulatory role in initiating asymmetric cell division, which must be maintained by the activity of aPKC [149,150].

T-cell generation from thymocytes in the thymus occurs through progressive spatiotemporal stages defined by changes in proliferation, differentiation, and cell death. Thymocytes interact with several distinct niche environments that influence the maturation process. Several recent studies have identified a role for asymmetric cell division in the niche, wherein cell specification is linked to unequal segregation of fate determinants [150,151]. Similar results have been seen with developing B-cells in germinal centers, sites within lymphoid organs where antibody producing cells mature [152,153]. Interestingly, molecular analysis of the polarity and spindle orientation complexes involved have revealed the use of several components conserved in many other models of asymmetric cell division, including Pins, Insc, Par/aPKC, Scribbled, Lis-1, and Numb [150,151].

## 5. Emerging Evidence for Centrosome Asymmetry

Work over the past nearly two decades has firmly established the importance of the mitotic apparatus in the asymmetric segregation of fate-determining polarity complexes within daughter cells. More recent studies have identified an additional level of asymmetry in several model systems, the asymmetric inheritance of mother and daughter centrosomes within progeny cells [154,155]. The centrosome serves as the primary microtubule organizing center during both interphase and mitosis and contributes to diverse biological processes beyond mitotic spindle assembly [156]. Centrosomes, which are an assembly of two barrel-shaped centrioles surrounded by a protenacious pericentriolar material, duplicate exactly once throughout G1/S/G2 and ultimately separate to initiate formation of the bipolar spindle during mitosis; defects in this precise duplication process are correlated with aneuploidy and tumorigenesis. Due to the sequential events of centriole disengagement, centriole duplication, centrosome maturation, and centrosome separation, the centriole pairs in each centrosome are morphologically and temporally asymmetric entities [154]. As a result, one daughter cell will inherit a centrosome containing the “older” mother centriole and the other the “younger”. Furthermore, proteomic analysis of centrosomal protein localization suggests some proteins asymmetrically concentrate at one centrosome [157]. Exciting discoveries have revealed this asymmetry correlates with distinct cell fate decisions during asymmetric cell division, in particular during stem cell divisions [155].

Following initial discovery and subsequent descriptions of asymmetric inheritance patterns of budding yeast spindle pole bodies [158,159], the first animal model system to establish biased centrosome segregation was the mGSC in *Drosophila*. In addition to niche-mediated spindle alignment described above, these cells preferentially deposit the “newer” daughter centrosome into the differentiating daughter cell, while the self-renewing stem cell retains the “older” mother centrosome (Figure 5A). The increased density of microtubules associated with the mother centrosome may allow it to more robustly interact with the polarity complex at the hub junction [160]. Interestingly, centrosome misorientation increases over the lifespan of the stem cell, resulting in cell-cycle arrest and ultimately a decline in spermatogenesis [161]. Studies in asymmetrically dividing neuroprogenitor cells of the mouse cortex revealed a striking parallel to the mGSC model—the mother and daughter centrosomes were biased to the progenitor and differentiated daughter cells, respectively, suggesting evolutionary conservation in asymmetric centrosome inheritance [162]. In *Drosophila* neuroblasts, however, although centrosome inheritance has a functional bias, it is the daughter centrosome that remains within the self-renewing neural stem cell with the differentiating cell inheriting the mother centrosome [163,164] (Figure 2A).

Recent studies have also begun to define the molecular basis of asymmetric centrosome establishment, including activities of Centrobin (an asymmetrically localizes centrosomal protein), Polo kinase, Pericentrin-like protein, and Cep135 [165,166]. These agents coordinately control asymmetric accumulation and retention of percentriolar material (PCM) on mother and daughter centrosomes. The resulting centrosomal asymmetry is crucial for proper centrosome and spindle orientation [164,165,167,168]. In mouse seminiferous tubules, Polo and Aurora-A kinases are assembled preferentially at the mother centrosome by the scaffolding protein, Gravin. Defects in the macromolecular complex results in defective spindle orientation and correlates with germ-line derived tumors [169]. Overall, these studies illustrate that non-random centrosome segregation occurs in diverse stem cell populations and across animal taxa. The exact biological reason(s) for biased centrosome inheritance, and whether centrosome identity directly participates in the cell fate decision process, remains to be fully answered.

## 6. Conclusions

Making correct cell fate decisions fundamentally contributes to both the developmental and homeostasis of complex tissue structures in multicellular organisms. Incorrect fate specification can lead to defects in this process and correlates with several abnormalities, including tumorigenesis. Cell fate acquisition is determined by the unequal distribution of fate-determining protein complexes into daughter cells during mitosis. Coordinated links between cortical polarity and mitotic spindle orientation underlie the cell**’**s ability to generate asymmetric daughter fates. Continued efforts to define the molecular mechanisms of asymmetric cell division will further illuminate this fascinating biological process and could open new avenues for therapeutic approaches to several human diseases. Surely additional regulatory mechanisms remain to be discovered, including how cell polarity and spindle positioning are coordinated with the cell cycle. Understanding the role of spindle orientation in human disease, and to what extent its role is causative in nature, will be another important area of continued research. Also of notable interest will be further understanding of potential cell-specific consequences of defective spindle orientation, for example in stem cells compared to non-stem cells.
